# Evaluation of a combined detection of SARS-CoV-2 and its variants using real-time allele-specific PCR strategy: an advantage for clinical practice

**DOI:** 10.1017/S095026882300184X

**Published:** 2023-11-24

**Authors:** Lucía Chaves-Blanco, Adolfo de Salazar, Ana Fuentes, Laura Viñuela, Javier Perez-Florido, Joaquín Dopazo, Federico García

**Affiliations:** 1 Servicio de Microbiología, Hospital Universitario Clínico San Cecilio, Granada, Spain; 2 Instituto de Investigación Biosanitaria Ibs.Granada, Granada, Spain; 3 Centro de Investigación Biomédica en Red en Enfermedades Infecciosas (CIBERINFEC), ISCIII, Madrid, Spain; 4 Computational Medicine Platform, Andalusian Public Foundation Progress and Health-FPS, Sevilla, Spain; 5 Institute of Biomedicine of Seville, IBiS, University Hospital Virgen del Rocío/CSIC/University of Sevilla, Sevilla, Spain; 6 FPS/ELIXIR-ES, Fundación Progreso y Salud (FPS), CDCA, Hospital Virgen del Rocio, Sevilla, Spain

**Keywords:** real-time allele-specific, SARS-CoV-2, surveillance

## Abstract

This study aimed to assess the ability of a real-time reverse transcription polymerase chain reaction (RT-PCR) with multiple targets to detect SARS-CoV-2 and its variants in a single test. Nasopharyngeal specimens were collected from patients in Granada, Spain, between January 2021 and December 2022. Five allele-specific RT-PCR kits were used sequentially, with each kit designed to detect a predominant variant at the time. When the Alpha variant was dominant, the kit included the HV69/70 deletion, E and N genes. When Delta replaced Alpha, the kit incorporated the L452R mutation in addition to E and N genes. When Omicron became dominant, L452R was replaced with the N679K mutation. Before incorporating each variant kit, a comparative analysis was carried out with SARS-CoV-2 whole genome sequencing (WGS). The results demonstrated that RT-PCR with multiple targets can provide rapid and effective detection of SARS-CoV-2 and its variants in a single test. A very high degree of agreement (96.2%) was obtained between the comparison of RT-PCR and WGS. Allele-specific RT-PCR assays make it easier to implement epidemiological surveillance systems for effective public health decision making.

## Introduction

Since severe acute respiratory syndrome virus (SARS-CoV-2) was first detected in 2019 [1], the virus has spread globally. Current estimates [2] show that SARS-CoV-2 has infected more than 674,000,000 persons and caused more than 6,860,000 deaths across the globe. The extreme high rate of transmission, and the event of prolonged infection in some vulnerable populations, such as the immunosuppressed patients [3], has prompted the accumulation of different nucleotide mutations in its genome, leading to the establishment of different viral lineages. Due to the impact of these variants on public health, the World Health Organization (WHO) proposed their classification as: Variants of Interest (VOI), Variants of Concern (VOC), Variants under Monitoring (VUM), and De-escalated variants. As of 23 February 2023, three Omicron lineages (BA.2, BA.4, and BA.5) are designated as VOC, while Alpha (B.1.1.7), Delta (B.1.617.2), and Omicron (BA.1) have been designated as De-escalated variants [4].

For Delta and Omicron, a great number of lineages, sublineages and recombinants [5–7] have been described; lineages, variants, subvariants, and recombinants are important as they may impact susceptibility to the host immune response and the severity of the infection, and may increase transmissibility of the virus and reduce the effectiveness of treatments [8–9].

Genomic surveillance of SARS-CoV-2 is essential for public health, as it is critical for decisions concerning diagnosis, therapy, and vaccination [10]. Genomic surveillance is critical to monitor the progression of the pandemic, and to adopt measures to minimize the number of people affected, hospital admissions, and the spread of the virus. For these reasons, to implement epidemiological surveillance systems that rapidly identify new SARS-CoV-2 variants is key.

Whole genome sequencing (WGS) is reference for the characterization of SARS-CoV-2 variants; although simplified protocols for WGS of SARS-CoV-2 are available [11], tests are still laborious, time consuming, and costly; most importantly, results are usually one to two weeks delayed, which is appropriate for surveillance but is insufficient for taking clinical decisions, especially for the use of monoclonal antibodies [12]. In this sense, real-time reversed transcribed PCR (RT-PCR) with multiple targets has emerged as an alternative that allows the rapid identification of key mutations in the Spike gene, which may be indicative of specific variants [13].

RT-PCR allows to obtain and report the results on the same day, and may be easily implemented in the routine of a clinical microbiology laboratory [14;15]. In this paper, we aimed to demonstrate the ability of RT-PCR to detect SARS-CoV-2 and its variants in a single test, increasing the number of samples that can be analysed.

## Materials & methods

For this study, we analysed nasopharyngeal specimens sent to Hospital Universitario Clínico San Cecilio, Granada, Spain, for SARS-CoV-2 detection between January 2021 and December 2022. Nasopharyngeal swabs were collected and transported in Universal Transport Medium (Vircell, Granada). Variant screening by RT-PCR and SARS-CoV-2 WGS was implemented in January 2021.

Viral RNA was extracted from 300 μl of sample using two different magnetic bead-based platforms according to manufacturers’ instructions: Maelstrom 9600/4800 (Taiwan Advanced Nanotech Inc, Taoyuan, Taiwan) and MagNA Pure Compact (Roche Diagnostics, Basel, Switzerland). After extraction, 40 μl of eluate was collected.

During the study period, five allele-specific RT-PCR kits (Vircell, Granada) were sequentially incorporated for the diagnosis of SARS-CoV-2 and real-time variant detection. Each kit was implemented according to the predominant variant (Table 1). When Alpha variant was the predominant VOC, the kit used incorporated HV69/70 deletion, as well as the E and N genes. At the time of the replacement of the Alpha variant by Delta, a kit that included L452R mutation, in addition to the E and N genes was used. When Omicron became dominant, L452R was replaced by N679K mutation. Later, to discriminate BA.1/BA.2 subvariants, the assay was modified to include the HV69/70 deletion, in addition to the N679K mutation. Finally, during the BA.4/BA.5 predominance period, a kit including N679K mutation, HV69/70 deletion, L452R mutation and the N gene was used. All tests were carried out on the CFX 96 (Bio-Rad, USA) according to manufactures instructions.

**Table 1.** Targets and implementation period of each RT-PCR assay

Targets	Period of use	Variant
N, E + AH69-70	1/01/21–14/07/21	Alpha
N, E + L452R	13/07/21–27/01/22	Delta
N, E + N679K	12/01/22–6/05/22	Omicron
N, E + N679K + AH69-70	7/05/22–31/07/22	Omicron subvariants BA.1/BA.2
N + N679K + AH69-70 + L452R	1/08/22–31/12/22	Omicron subvariants BA.1/BA.2/BA.4/BA.5

All samples in the study were SARS-CoV-2 positive: 39,466 were samples for which variant testing was available; from these, WGS was available from data of Andalusian Genomic Surveillance Circuit in 1896 samples, with a cycle threshold value <33. Sequencing of SARS-CoV-2 genome was performed according to the modified Artic Network protocol. Libraries were prepared with the CovidSeq kit (Illumina Inc, USA) according to the manufacturer instructions, and were sequenced using Illumina Miseq or llumina Nextseq 1000. Analysis included generation of SARS-CoV-2 consensus sequence (viralrecon), mutation detection (nextclade) and lineage designation (pangolin) according to the workflow established in the Andalusian Genomic Surveillance Circuit [16].

Sensitivity, specificity, positive predictive value (PPV), and negative predictive value (NPV) were used to define the accuracy of RT-PCR, using WGS as reference. The corresponding two-tailed 95% score (Wilson) confidence intervals (CIs) were also estimated.

The study was approved by Hospital Universitario Clínico San Cecilio review board. Given the deidentified nature of testing, individual patient consent was not required for this study.

## Results

**Figure 1.**
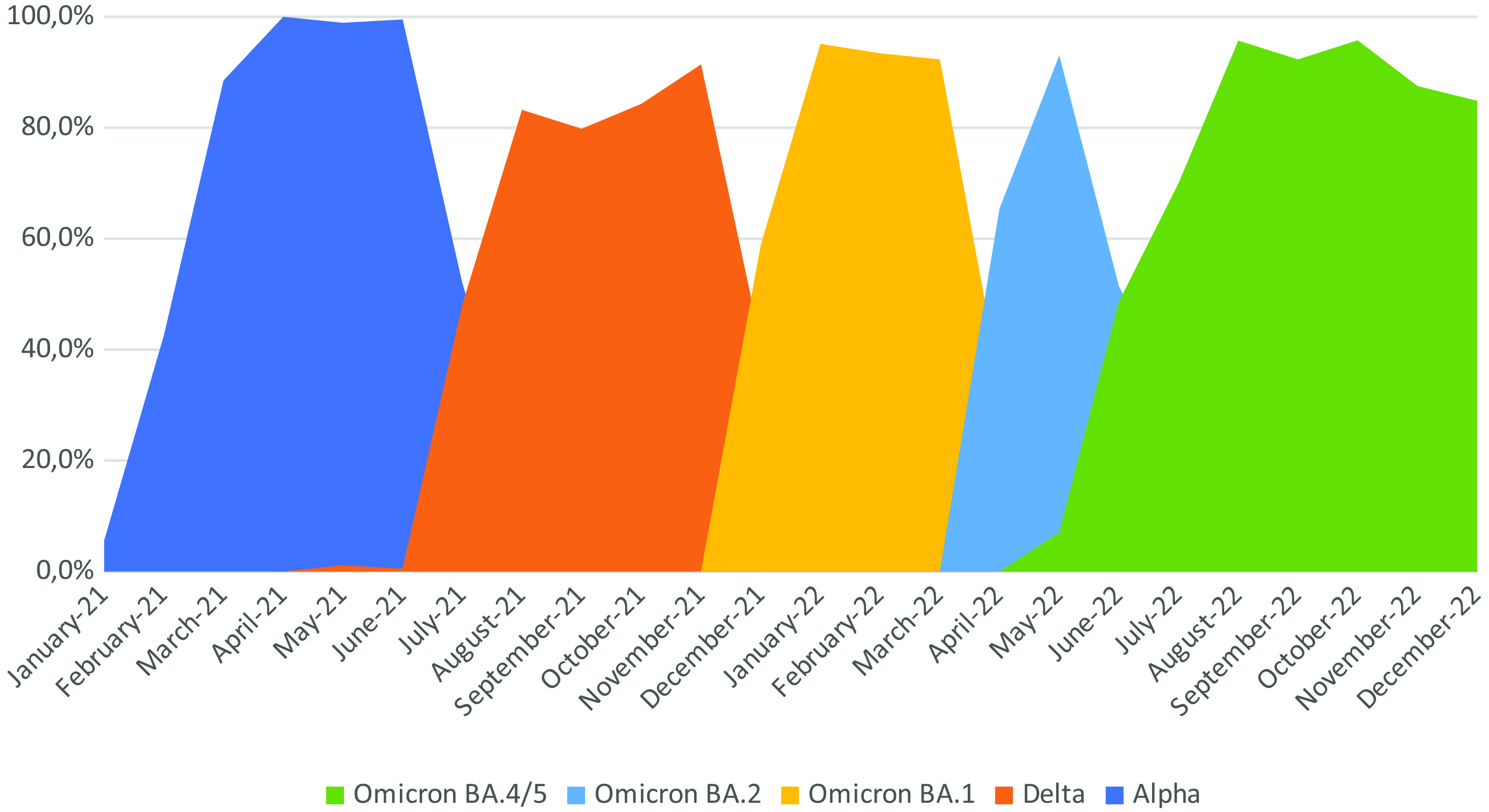
Evolution of variants detected by the RT-PCR allele-specific assays.

Variant screening results at the time of diagnosis was available for 39,466 SARS-CoV-2 positive patients. Alpha was first detected in January 2021 and by April 2021 had fully replaced the prior variant. By May 2021 Alpha begun to be replaced by Delta, and by the time we implemented the Delta assay (mid-July 2021) this variant already represented 50%. Delta continued to rise to almost 100% until November 2021 and was very quickly almost fully replaced by Omicron by January 2022, when Omicron assay was implemented in our laboratory. By April 2022, a new assay was introduced to detect Omicron subvariants, BA.1 and BA.2. BA.1 was the predominant subvariant until March 2022 (92.3%) when BA.2 started to emerge (7.7%). BA.2 rapidly replaced BA.1, accounting for almost 100% of cases by May 2022. By July 2022, BA.4 and BA.5 had replaced almost 50% of BA.2 and in August 2022 we implemented a variant assay that could discriminate BA.1, BA.2, and BA.4/5. The latter was already 95% prevalent and remained so until the end of the study period. These data are presented in Figure 1.

Whole genome sequencing of SARS-CoV-2 was also available, as a part of the Andalusian and Spanish surveillance programme in 1896 samples and were therefore available for assay comparison. Two hundred and twenty-three samples were compared for Alpha, 492 for Delta, 590 for Omicron, 623 for Omicron subvariants BA.1/BA.2 and 300 for BA.1/BA.2/BA.4/BA.5. Overall concordance was 96.2% (95 CI 95.1–97.1), showing a Kappa agreement of 0.87 (95% CI 0.84–0.90). A head-to-head comparison between RT-PCR and WGS is shown in Table 2. Sensitivity of the RT-PCR variant assays ranged from 92.3% for the BA.1/BA.2 assays to 99.8% for Omicron. Specificity, as well as PPV, was 100% for all the variant assays investigated. NPV was ranged from 82.7% for BA.1/BA.2 to 99.4% for the Omicron and BA.1/BA.2/BA.4/BA.5 assays.

**Table 2.** Head-to-head comparison between RT-PCR and WGS

VOC			WGS					
			Positive	Negative	Sensitivity (95 % CI)	Specificity (95 % CI)	PPV (95 % CI)	NPV (95 % CI)
RT-PCR	Alpha	Positive	55	0	96.5%	100%	100%	98.8%
		Negative	2	166^a^	(87.9%–99.6%)	(97.8%–100%)		(95.5%–99.7%)
	Delta	Positive	226	0	91.9%	100%	100%	92.5%
		Negative	20	246^b^	(87.7%–95%)	(98.5%–100%)		(89.0%–94.9%)
	Omicron	Positive	420	0	99.8%	100%	100%	99.4%
		Negative	1	169^c^	(98.7%–100%)	(97.8%–100%)		(96.0%–99.9%)
	BA.1/BA.2	Positive	422	0	92.3%	100%	100%	82.7%
		Negative	35	166^a^	(89.5%–94.6%)	(97.8%–100%)		(77.6%–86.8%)
	BA.1/BA.2/BA.4/BA.5^d^	Positive	133	0	99.3%	100%	100%	99.4%
		Negative	1	166^a^	(95.9%–99.9%)	(97.8%–100%)		(95.9%–99.9%)

*Note*: For sensitivity, specificity, and positive and negative predictive values, WGS was considered as reference.

a AY.4, *n* = 59; AY.5, *n* = 3; AY.7, *n* = 1; AY.9, *n* = 37; AY.12, *n* = 18; B.1.617.2, *n* = 48.

b BA.1, *n* = 243; BA.2, *n* = 3.

c AY.4, *n* = 59; AY.5, *n* = 3; AY.7, *n* = 1; AY.9, *n* = 37; AY.12, *n* = 18; B.1.617.2, *n* = 51.

d BA.2, *n* = 4; BA.2.75, *n* = 8; BA.5, *n* = 122.

## Discussion

Whole genome sequencing is the gold standard for genomic surveillance of SARS-CoV-2. However, as it requires highly skilled professionals and is laborious, time consuming, and expensive, during COVID-19 pandemic many countries have had difficulties to meet international recommendations [17;18]. In this study, we describe an allelic specific RT-PCR strategy used to identify SARS-CoV-2 variants at the time of diagnosis. We show that this strategy can detect variants with high accuracy, and a high degree of concordance to WGS and, most importantly, allows their detection at the time of diagnosis, which is especially relevant for certain therapeutic strategies such as the treatment with monoclonal antibodies.

In our study, we made a head-to-head comparison of the RT-PCR variants assays with WGS. Previous studies [15;19] have also explored the use of variant detection by RT-PCR from nasopharyngeal and from waste-water, with high levels of concordance. However, our study provides new information and insights of special importance for patient management: this is the first study to use variant assays for both SARS-CoV-2 diagnosis and variant identification, vital for treatment decisions, especially in severe cases where certain Omicron variants may resist monoclonal antibodies [12;20]; we also, showed excellent assay specificity and positive predictive values, pivotal for clinical decisions; finally, our data support these assays to bolster regional and national variant surveillance, particularly beneficial for low- and middle-income countries with limited access to whole genome sequencing.

Our study’s main limitation is the different sample size for the different variant assays evaluated throughout the study period. As SARS-Cov-2 WGS was implemented at our centre at the beginning of the Alpha wave in Spain, our capability for WGS during the first stages of the pandemic was lower, hence limiting the number of positive samples that could be evaluated.

In summary, we show for the first time to our knowledge, a longitudinal evaluation and comparison of RT-PCR to diagnose SARS-CoV-2 and assign Alpha, Delta, and Omicron variants, and Omicron BA.1/BA.2/BA.4/BA.5 sublineages, at the time of diagnosis on the same assay, showing excellent specificity and positive predictive values, when compared to whole genome sequencing. These assays provide reliable and timely information on SARS-CoV-2 variants that may be used for taking clinical decisions, especially to decide on the use of monoclonal antibodies. Finally, we believe that these assays may also be used in low- and middle-income countries to provide preliminary results for genomic surveillance.

## Data Availability

The datasets generated during the current study are available from the corresponding author on reasonable request.
